# Comparison of anther transcriptomes in response to cold stress at the reproductive stage between susceptible and resistant *Japonica* rice varieties

**DOI:** 10.1186/s12870-022-03873-6

**Published:** 2022-10-26

**Authors:** Zhenhua Guo, Wendong Ma, Lijun Cai, Tao Guo, Hao Liu, Linan Wang, Junliang Liu, Bo Ma, Yanjiang Feng, Chuanxue Liu, Guojun Pan

**Affiliations:** 1 Rice Research Institute of Heilongjiang Academy of Agricultural Sciences, 154026 Jiamusi, Heilongjiang China; 2 National Engineering Research Center of Plant Space Breeding, South China Agricultural University, 510642 Guangzhou, Guangdong China; 3 Jiamusi Branch of Heilongjiang Academy of Agricultural Sciences, 154007 Jiamusi, Heilongjiang China; 4 Jiamusi Longjing Seed Industry Co., LTD, 154026 Jiamusi, Heilongjiang China; 5 Crops Research Institute, Guangdong Academy of Agricultural Sciences, 510640 Guangzhou, Guangdong China; 6 Qiqihar Branch of Heilongjiang Academy of Agricultural Sciences, 161006 Qiqihar, Heilongjiang China

**Keywords:** Rice, Cold stress, Reproductive stage, Transcriptome analysis, Plant hormone

## Abstract

**Background:**

Rice is one of the most important cereal crops in the world but is susceptible to cold stress (CS). In this study, we carried out parallel transcriptomic analysis at the reproductive stage on the anthers of two *Japonica* rice varieties with contrasting CS resistance: cold susceptible Longjing11 (LJ11) and cold resistant Longjing25 (LJ25).

**Results:**

According to the obtained results, a total of 16,762 differentially expressed genes (DEGs) were identified under CS, including 7,050 and 14,531 DEGs in LJ25 and LJ11, respectively. Examining gene ontology (GO) enrichment identified 35 up- and 39 down-regulated biological process BP GO terms were significantly enriched in the two varieties, with ‘response to heat’ and ‘response to cold’ being the most enriched. Kyoto Encyclopedia of Genes and Genomes (KEGG) analysis identified 33 significantly enriched pathways. Only the carbon metabolism and amino acid biosynthesis pathways with down-regulated DEGs were enriched considerably in LJ11, while the plant hormone signal transduction pathway (containing 153 DEGs) was dramatically improved. Eight kinds of plant hormones were detected in the pathway, while auxin, abscisic acid (ABA), salicylic acid (SA), and ethylene (ETH) signaling pathways were found to be the top four pathways with the most DEGs. Furthermore, the protein-protein interaction (PPI) network analysis identified ten hub genes (co-expressed gene number ≥ 30), including six ABA-related genes. Various DEGs (such as *OsDREB1A*, *OsICE1*, *OsMYB2*, *OsABF1*, *OsbZIP23*, *OsCATC*, and so on) revealed distinct expression patterns among rice types when the DEGs between LJ11 and LJ25 were compared, indicating that they are likely responsible for CS resistance of rice in cold region.

**Conclusion:**

Collectively, our findings provide comprehensive insights into complex molecular mechanisms of CS response and can aid in CS resistant molecular breeding of rice in cold regions.

**Supplementary Information:**

The online version contains supplementary material available at 10.1186/s12870-022-03873-6.

## Background

As a primary carbohydrate source, rice (*Oryza sativa* L.) feeds more than 50% of the world’s population and provides more than 20% of the caloric intake of each person in the world [1, 2]. Rice is susceptible and highly vulnerable to CS since it originated in tropical or sub-tropical areas. Low temperatures are the primary constraint on its production across the globe, particularly in upland and deep-water settings [3]. At present, more than 24 countries containing approximately 15 million hm^2^ of rice fields are vulnerable to CS, especially in Japan, Korea, Australia, and China [4, 5]. In China, injury due to CS can lead to an annual loss of about 3–5 million tons in rice production [6]. Rice cold-resistant breeding is an effective technique to deal with these conditions and ensure safe rice production in sensitive areas owing to the unpredictable cold weather that occurs regularly due to catastrophic climate disasters.

Rice is highly susceptible to CS throughout its life cycle. Rice demonstrates delayed or reduced germination rates when exposed to CS during the germination stage. Low temperatures during the seedling stage can cause leaf yellowing and curling, shorter plants, and reduced tillering [7]. However, rice is most susceptible to CS at the reproductive stage compared to other periods since CS at the reproductive stage directly and adversely affects pollen fertility, seed set, and grain yield [8, 9]. Furthermore, during the reproductive stage, the transition of the tetrad to the young microspore stage (YM stage, also called the early uni-nucleate stage) is crucial to normal pollen formation, which is most susceptible to low temperatures [5, 10]. Previous studies have shown that down-regulation of *OSINV4*, one of the cell wall-bound invertase encoding genes, inhibits its activity and increases the sucrose content in the anthers [11]. Subsequently, the tapetal cells are hypertrophied according to the increased sucrose content resulting in the final degeneration of the microspores [9, 12]. *Osg1* is a *β-*1,3-glucanase encoding gene that is also related to pollen fertility in rice since its suppression during the YM stage hinders pollen development leading to an increase in the number of sterile pollen grains [13, 14].

The physiological and biochemical characteristics changed along with the morphological and histological properties [15]. Such as phytohormones, amino acids, and reactive oxygen species (ROS) are CS-related and may be treated as indicators of CS [16]. The plant cell membranes usually become more rigid, and their physical phases change after sensing the CS when exposed to low temperatures. The CS signal is then transmitted to calcium ion (Ca^2+^), which acts as a secondary messenger for activating the DREB-CRT/DRE pathway and inducing the expression of cold-responsive genes based on its concentration changes in the cytosol [17–19]. ABA is a phytohormone that is crucial for abiotic stress response in rice and plays an essential role in signaling cascades to activate cold-regulated genes downstream through Ca^2+^ induction via ABA-dependent or ABA-independent pathways [20, 21]. During the CS response, some metabolites (i.e., sugar and proline) are accumulated as osmolytes and cryoprotectants to prevent damage [22–24]. At the same time, other signaling molecules, including ROS, activate the MAPK cascade pathway [25, 26]. The phytohormones, which are crucial for plant growth and abiotic stress responses, are also present. These include ETH, indoleacetic acid (IAA), salicylic acid (SA), and jasmonic acid (JA). However, they are most needed in trace amounts [27, 28]. *OsBIERF1*, *OsBIERF3*, and *OsBIERF4* are ETH response transcription factors induced by salt, cold, and drought stresses [29].

Previous studies have shown that some genes related to pollen development are involved in CS response at the reproductive stage [11, 22, 30, 31]. As a conserved leucine-rich repeat receptor-like kinase encoding gene, *CTB4a* positively regulates CS tolerance at the reproductive stage. Its overexpression can also increase ATP synthase activity and ATP content and improve pollen fertility, seed setting rate, and rice yield under CS at the reproductive stage [32]. Earlier transcriptome investigations of anthers demonstrate that gene expression is dramatically affected by CS during the reproductive stage, with several genes involved in carbohydrate metabolism, signal transduction, ion transport, lipid metabolism, and transcriptional regulation being disrupted [5, 9, 33, 34]. However, the complex genetic and molecular mechanisms involved in CS tolerance of rice are not fully understood to date. In this study, we performed parallel transcriptomic analysis on anthers in two *Japonica* rice varieties, LJ11 and LJ25, at the reproductive stage to characterize the DEGs involved in cold resistance and understand the molecular mechanisms for CS response in rice. The findings provide extensive insights into transcriptional regulation during CS at the reproductive stage in rice, which aid in the efforts for molecular breeding of CS-resistant rice cultivars in the future.

## Results

### Morphological and physiological characteristics of contrasting genotypes under CST

**Fig. 1:**
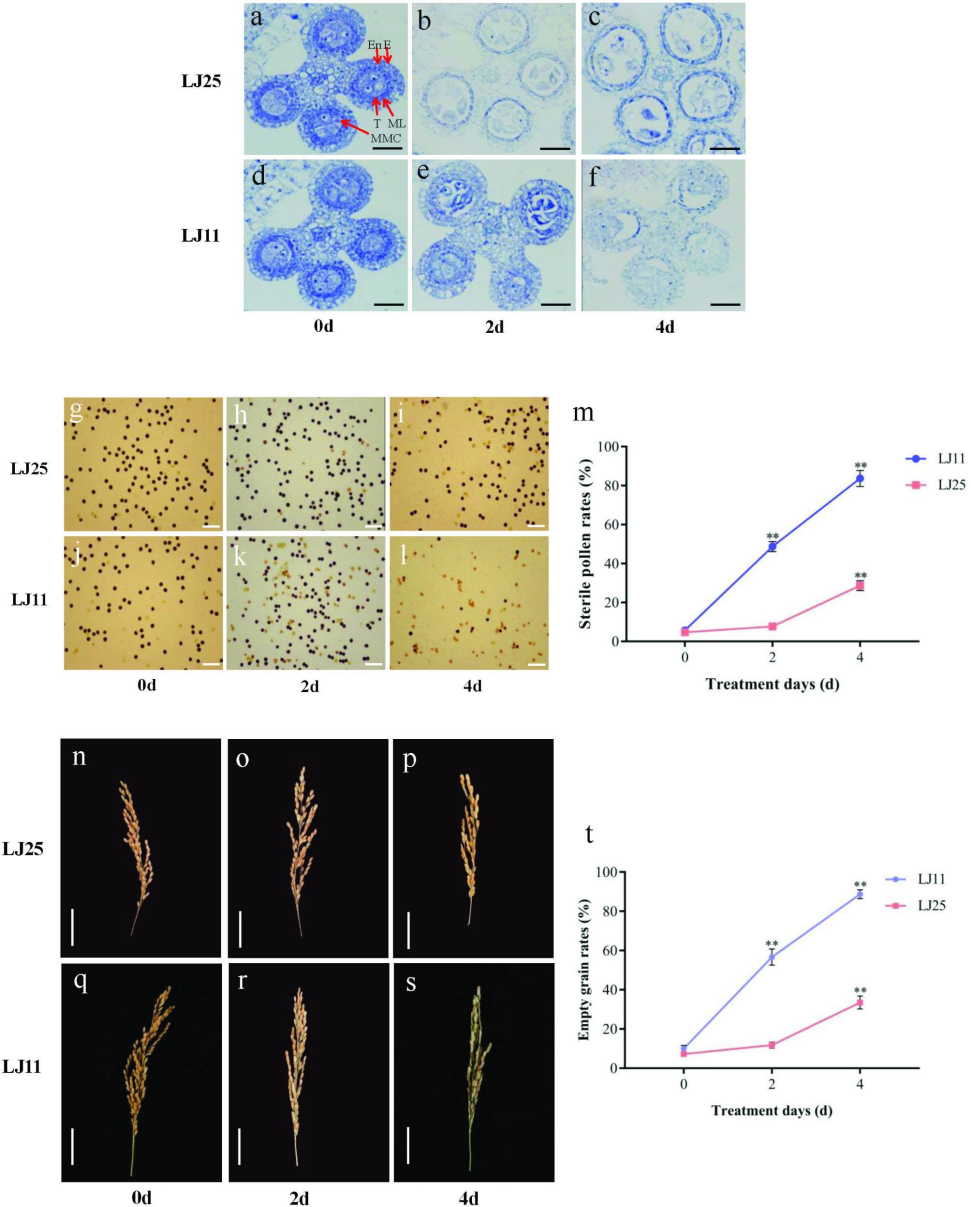
Morphological and physiological characteristics of contrasting genotypes under CST. **(a-c) **bold the panel labels a-c here Cytological observation of the anther development in LJ25 after 0, 2, and 4 days of CST. **(d-f)** bold the panel labels d-f here Cytological observation of the anther development in LJ11 after 0, 2, and 4 days of CST. Scale bar = 100 mm. E, epidermis; En, endothecium; ML, middle layer; ML, middle layer; T, tapetum; MMC, microspore mother cell. **(g-i) **bold the panel labels g-i here Pollen fertility of LJ25 at 0, 2, and 4 days of CST. **(j-l)** bold the panel labels j-l here Pollen fertility of LJ11 at 0, 2, and 4 days of CST. Scale bar = 100 mm. **(m)** bold the panel label m here Sterile pollens rates of LJ25 and LJ11 under CST. The x-axis represents the number of days of CST. **(n-p) **bold the panel labels n-p here LJ25 panicles at 0, 2, and 4 days of CST. **(q-s)** bold the panel labels q-s here LJ11 panicles at 0, 2, and 4 days clustering analysisof CST. Scale bar = 5 cm. **(t)** bold the panel label t here Empty grain rates of LJ25 and LJ11 under CST. The x-axis represents the number of days of CST. ** represent p < 0.01. Error bars represent the SD

Anthers of the two varieties were obtained during various CST periods. The cellular structures were observed via transverse semi-thin sections to examine impact of CS on pollen formation at the cytological level. In the control group (zero days of CST), the anthers of LJ11 and LJ25 were both in the pre-meiosis stage, and no noticeable morphological differences were observed. Four distinct layers of cells were observed in the anther wall, which from the outside was composed of normal epidermis, endothecium, a middle layer, and tapetum. Each layer had a thick cytoplasm and regular cell characteristics (Fig. 1a and d). After two days of CST, the pollen mother cells in the LJ25 anther entered the meiosis stage, the cells in the middle layer of the anther wall became thinner, and the cytoplasm of the tapetal cells was concentrated and deeply dyed. LJ11, however, was significantly affected by CS. The anthers of LJ11 were still at the stage of pollen mother cells, but the cytoplasm of tapetal cells also began to concentrate, and the staining was also deepened (Fig. 1b and e). The LJ25 anther cells were still in the meiosis stage at four days of CST. The tapetal cells continued to concentrate, and staining deepened. However, the anthers of LJ11 were still in the stage of pollen mother cells, the cytoplasm of tapetal cells continued to concentrate, the staining deepened, and part of the tapetal disintegrated and fractured. The pollen mother cells of LJ11 were obviously squeezed and vacuolated (Fig. 1c and f). These results suggest that the male sterility of LJ11 was associated with abnormality degeneration of the tapetum and the pollen mother cells.

Only a few sterile pollen grains were observed in both varieties without CST, according to the pollen fertility investigation of LJ11 and LJ25 at various CS phases (4.67% in LJ25 and 5.67% in LJ11) (Fig. 1 g, 1j, 1 m, and Table 1). After two days of CST, the sterile pollen rates of LJ11 significantly increased (48.67%), while no significant increase was detected in LJ25 (7.67%) (Fig. 1 h, 1k, 1 m, and Table 1). Moreover, up to the end of four days of CST, most of the pollens in LJ11 were sterile (83.67%, significantly increased), which was improved considerably in LJ25 (28.67%) (Fig. 1i L, 1 m and Table 1).

**Table 1:** Sterile pollen rates of contrasting genotypes under CS treatment

Treatment days	Sterile pollen rates (%)					
	**LJ11**			**LJ25**		
0	4	6	7	4	6	4
2	51	46	49	9	6	8
4	85	87	79	26	29	31

After four days of continuous exposure to a low temperature of 12 °C, the two cultivars’ empty grain rates (EGRs) were calculated every two days during CST. LJ11 and LJ25 had EGRs of 9.95 and 7%, respectively, in the absence of CST (Fig. 1n, q and t, and Table 2). However, the EGRs significantly increased in LJ11 after two days of CST (56.65%), with almost all grains infertile after four days of the CST (88.70%) (Fig. 1r, s and t; Table 2). These findings imply that LJ11 was susceptible to low temperatures during the reproductive stage. There was no significant increase in EGRs in LJ25 after two days of CST relative to the control group (11.73%). Still, there was a considerable increase after four days of CST (33.48%), indicating that LJ25 may be resistant to CS during the reproductive stage (Fig. 1o, p and t, and Table 2).

**Table 2:** Empty grains rates of contrasting genotypes under CS treatment

Treatment days	Empty grain rates (%)											
	**LJ11**						**LJ25**					
0	8.9	10.1	7.7	12.3	9.5	11.2	5.2	6.2	8.8	6.9	8.3	7.6
2	53.2	59.7	55.2	60.1	50.9	60.8	10.3	11.6	12.2	13.6	12.8	9.9
4	88.1	90.8	91.1	86.5	89.9	85.8	28.9	35.7	32.3	36.5	30.8	36.7

### Analysis of RNA-seq and gene expression profiles

**Fig. 2:**
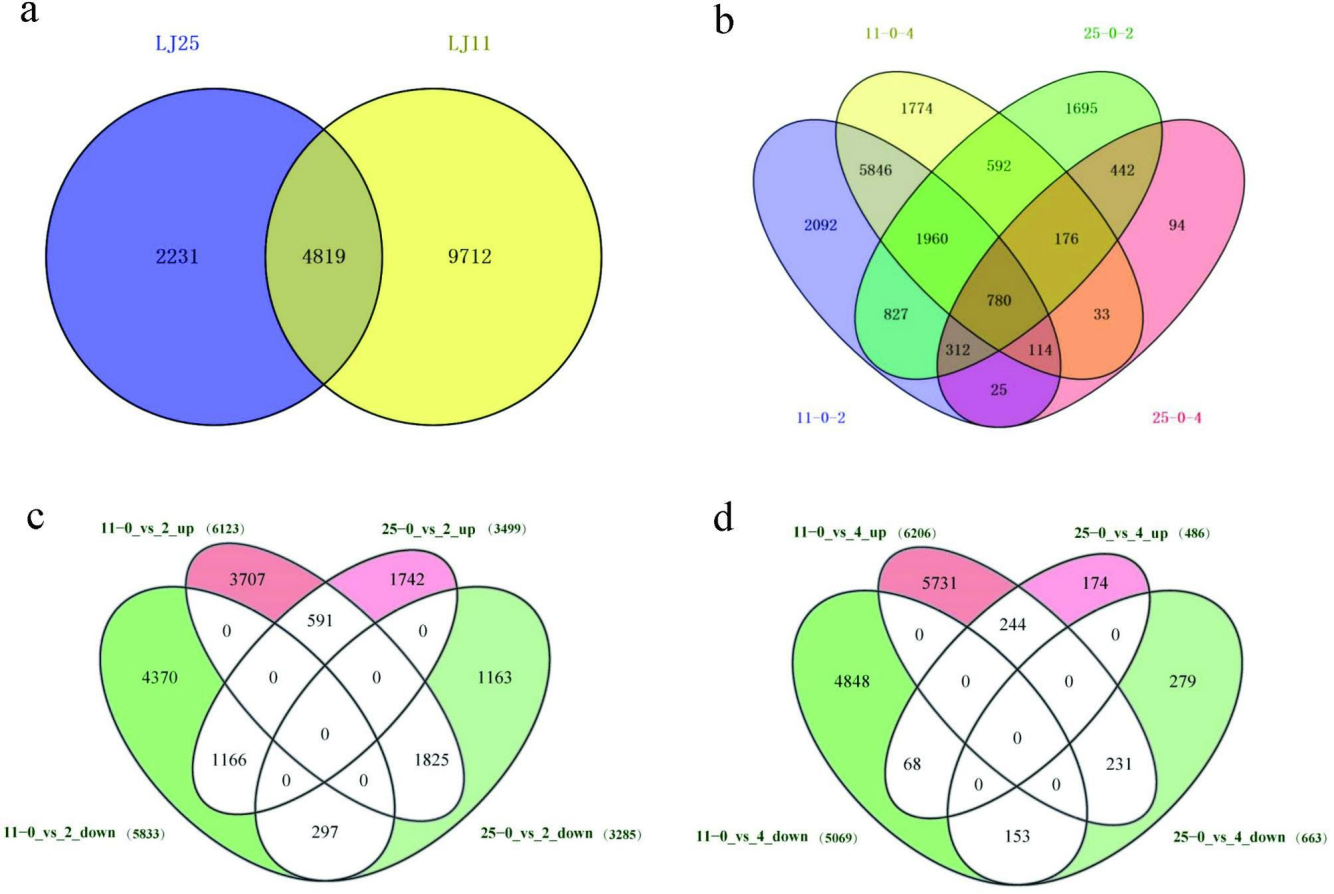
Statistical analysis of DEGs in LJ25 and LJ11 under the CST. (**a**) A Venn diagram depicting all DEGs detected as part of the CST between LJ25 and LJ11. (**b**) The DEGs under CST in the four compared groups between LJ25 and LJ11. (**c**) DEGs were detected after two days of CST between LJ25 and LJ11. (**d**) Venn diagram of the DEGs observed after four days of CST between LJ25 and LJ11. ‘11’ and ‘25’ represent LJ11 and LJ25, respectively. ‘0_vs_2’ and ‘0_vs_4’ represent two and four days of cold stress treatment, respectively. The numbers in brackets represent the DEG numbers. ‘Up’ and ‘down’ mean the up-and down-regulated DEGs. The numbers in different parts of the Venn diagram represent the numbers of DEGs specific or commonly identified in other regions

Eighteen libraries were constructed in triplicate using two cultivars and three CST periods (zero, two, and four days). In total, 156.02 GB of clean data (including 527.83 million clean reads) was generated through RNA-seq. Overall, 79.55–84.3% of high-quality reads in each sample were mapped to the rice reference genome with TopHat2 (Table 3) [35]. The samples in the same CST group (two and four days) from the same cultivar were clustered together, according to sample-to-sample clustering analysis (Figure. S1)link S1 accordingly, indicating remarkable repeatability between samples. With the thresholds of FC ≥ 2 and FDR ≤ 0.01, 16,762 DEGs were detected among the 18 samples under CS. Additionally, 7,050 and 14,531 DEGs were found in LJ25 and LJ11, respectively, while 4,819 DEGs were identified in both varieties following CST (Fig. 2a and Tables S3, S4link S3 and S4 accordingly). Similarly, after two days of CST, 11,956 and 6,784 DEGs were identified in LJ11 and LJ25, respectively, whereas after four days of CST, 11,275 and 1,149 DEGs were identified in LJ11 and LJ25, respectively (Fig. 2b and Tables S3, S4link S3 and S4 accordingly). Figure 2b demonstrates that after two and four days of CST, 2,092 and 1,774 DEGs were notably discovered in LJ11, while 1,695 and 94 DEGs were found in LJ25. In addition, 780 DEGs were found to be shared by the four groups that were compared. More specifically, DEGs were reclassified using the different Venn diagram portions, and 6,123 up- and 5,833 down-regulated DEGs were revealed in LJ11, whereas 3,499 up- and 3,825 down-regulated DEGs were found in LJ25 (Fig. 2c and d, and Table S5).

**Table 3:** Quality assessment of transcriptome data overview

Samples	Clean reads	Clean bases	Mapped Reads	Mapped Reads/Reference Genome (%)	GC Content (%)	%≥Q30
LJ25_0_1	21,609,792	7,980,503,202	36,434,612	84.30	55.53	92.43
LJ25_0_2	28,917,176	10,124,446,350	47,992,825	82.98	56.07	91.78
LJ25_0_3	30,826,689	9,235,905,684	51,148,445	82.96	55.56	91.98
LJ25_2_1	27,882,196	6,401,626,196	45,602,317	81.78	55.52	91.46
LJ25_2_2	27,757,905	10,975,096,620	45,293,095	81.59	54.52	91.74
LJ25_2_3	24,935,169	9,316,606,172	41,000,921	82.22	55.39	92.05
LJ25_4_1	30,249,660	9,115,767,834	49,312,142	81.51	55.28	91.94
LJ25_4_2	30,851,206	8,709,768,128	51,115,346	82.84	55.05	91.96
LJ25_4_3	34,697,837	7,971,386,384	56,124,178	80.88	55.67	91.3
LJ11_0_1	26,971,012	6,382,284,518	43,361,427	80.39	56.58	91.10
LJ11_0_2	34,230,674	8,551,868,430	55,243,173	80.69	56.40	91.52
LJ11_0_3	31,262,982	9,126,285,864	50,368,723	80.56	55.92	91.26
LJ11_2_1	21,610,740	8,250,744,030	34,915,829	80.78	54.77	91.39
LJ11_2_2	37,117,251	8,212,639,628	60,514,688	81.52	55.35	91.86
LJ11_2_3	31,497,458	7,351,457,562	50,799,160	80.64	55.02	91.29
LJ11_4_1	30,907,327	8,935,105,036	49,175,101	79.55	53.92	91.86
LJ11_4_2	29,528,812	9,108,001,686	48,432,689	82.01	53.98	91.92
LJ11_4_3	27,009,855	10,268,046,118	44,750,148	82.84	53.40	92.20

Several DEGs were found to have different expressed levels or patterns when the DEGs in the LJ11 and LJ25 were compared directly (Table S6), these includes *Os03g0315400* (*OsMYB2)* [36], *Os03g0820300* (*ZFP182*) [37], *Os09g0522200 (OsDREB1A)* [38], *Os02g0704000* (*OsNCED1)* [39], *Os03g0131200* (*OsCATC)* [40], *Os03g0401300* (*RSUS1)* [41], *Os11g0523700* (*OsICE1)* [42], *Os03g0285800* (*OsMAP1)* [43], *Os01g0867300* (*OsABF1)* [44], *Os03g0181100* (*OsJAZ10)* [45], *Os03g0180900* (*OsJAZ11)* [45], and so on.

### GO functional enrichment analysis

**Fig. 3:**
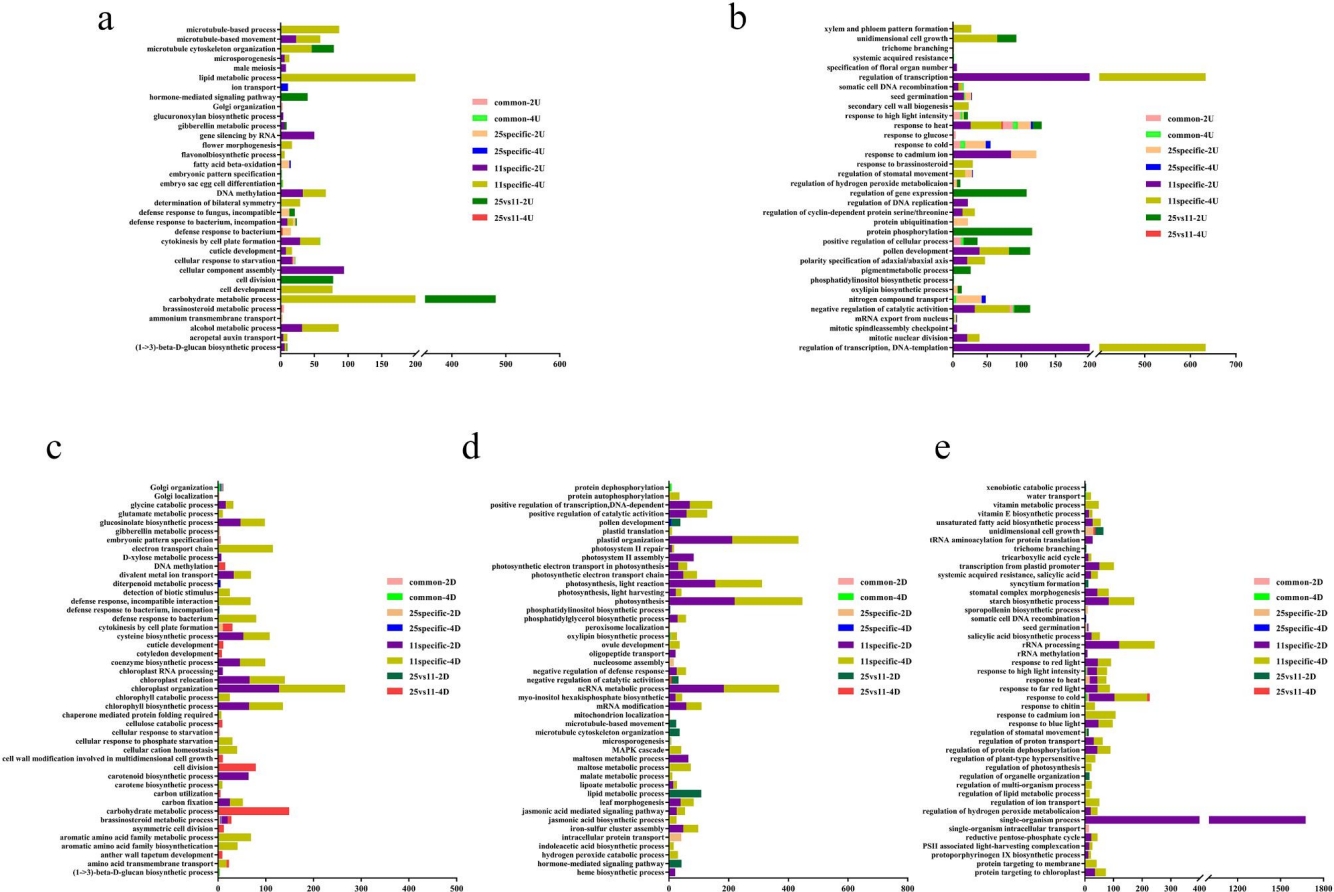
BPs GO enrichment analysis in LJ25 and LJ11 under cold stress. (**a**-**b**) BPs GO enrichment analysis of the up-regulated DEGs in the different parts of the Venn diagram (Fig. 2c) between LJ11 and LJ25. (**c**-**e**) BPs GO enrichment analysis on the down-regulated DEGs in the different parts of the Venn diagram (Fig. 2d) between LJ11 and LJ25. ‘Common’ denotes the DEGs that commonly exist in the two parts of the Venn diagram. ‘Specific’ shows the DEGs that only exist in one part of the Venn diagram. ‘25 vs. 11’ represents the DEGs that show opposite expression patterns between LJ25 and LJ11. ‘2U’ and ‘4U’ represent the up-regulated DEGs after two and four days of CST, respectively. ‘2D’ and ‘4D’ represent the down-regulated DEGs after two and four days of CST, respectively. The x-axis represents the numbers of DEGs involved in various GO terms

To further explore the biological functions of DEGs, GO enrichment analyses (especially in the ‘biological process’ (BP) category) were carried out within the different parts of the Venn diagrams (Fig. 2c and d) based on the threshold of KS (Kolmogorov-Smirnov) < 0.001. Among the up-regulated DEGs, 10 and 15, 15 and 11, 28 and 32, 13 and 4 BP GO terms were significantly enriched in the parts of common-2U and common-4U, 25 specific-2U and 25 specific-4U, 11 specific-2U and 11 specific-4U, and 25 vs. 11-2U and 25 vs. 11-4U, respectively, while, the most significantly enriched terms were secondary cell wall biogenesis (2.00E-08) in LJ11 after four days of CST (Fig. 3a-b and Table S7). In the down-regulated DEGs, 15 and 9, 12 and 6, 61 and 82, and 26 and 6 BP GO terms, were significantly enriched in the same comparison groups as the up-regulated DEGs (common-2D and common-4D, 25 specific-2D and 25 specific-4D, 11 specific-2D and 11 specific-4D, and 25 vs 11-2D and 25 vs 11-4D). The most significantly enriched term was chloroplast relocation (4.50E-29) in LJ11 after four days of CST (Fig. 3c-e and Table S8). 68 and 135 BP GO Terms were significantly enriched in the up and down-regulated DEGs, respectively (Table S9). The specifically up-regulated DEGs were primarily enriched in the regulation of transcription and carbohydrate metabolic process in LJ11 after four days of CST, including 365 and 333 DEGs respectively. In LJ25, the BPs with the most DEGs primarily included the response to cadmium ion and nitrogen compound transport after two days of CST, each with 37 DEGs. Moreover, among the different parts of the Venn diagram, the most enriched categories were response to heat and cold (Fig. 3 and Tables S7-9).

### KEGG pathway enrichment analysis

**Fig. 4:**
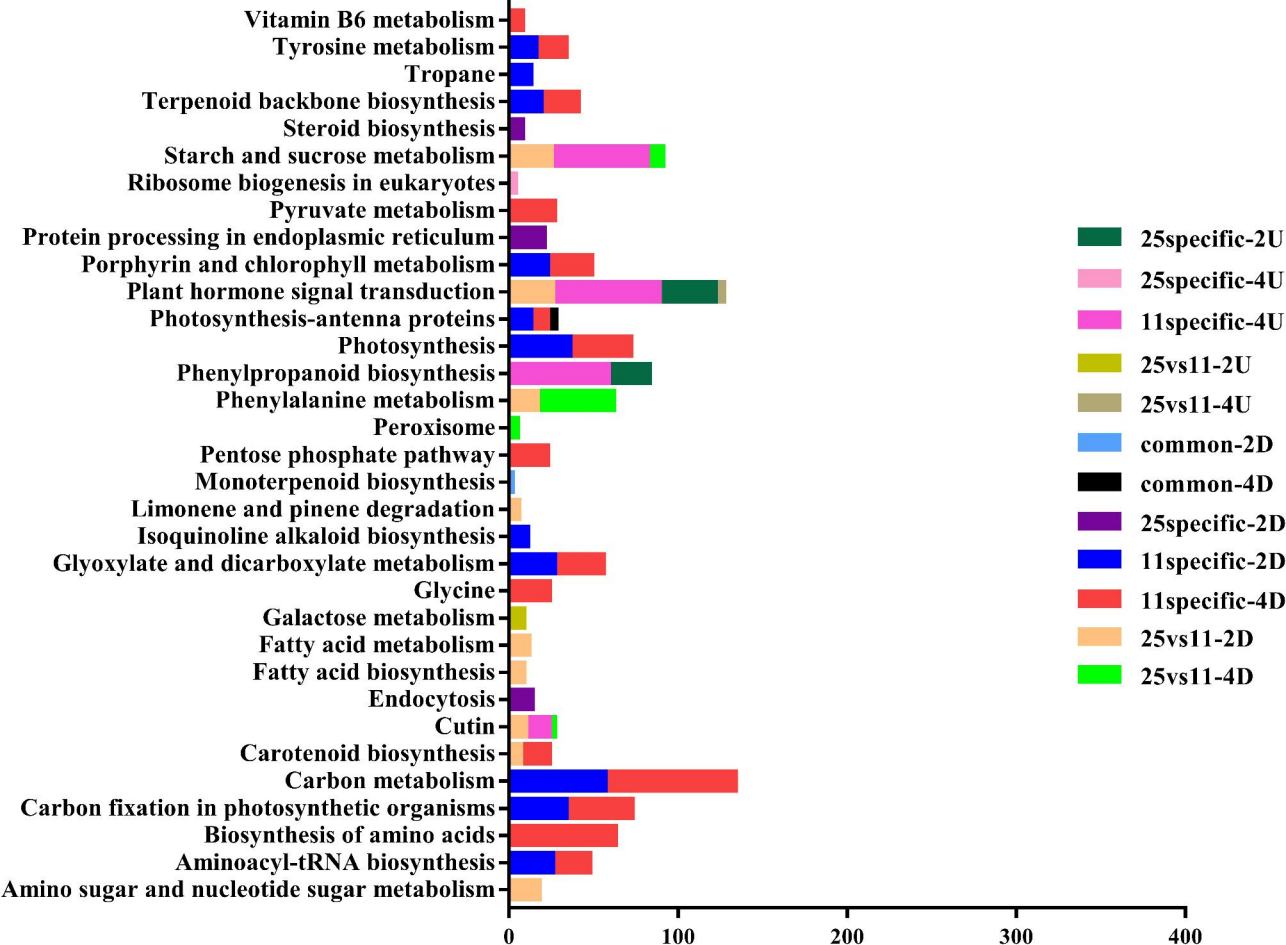
KEGG pathway enrichment analysis of total DEGs at different parts in the Venn diagram under CST between LJ25 and LJ11. ‘Common’ represents the DEGs that commonly exist in the two parts of the Venn diagram. ‘Specific’ signifies the DEGs that only exist in one part of the Venn diagram. ‘25 vs 11’ denotes the DEGs that show opposite expression patterns between LJ25 and LJ11. ‘2U’ and ‘4U’ represent the up-regulated DEGs after two and four days of CST, respectively. ‘2D’ and ‘4D’ characterize the down-regulated DEGs after two and four days of CST, respectively. The x-axis represents the numbers of DEGs involved in various KEGG pathways

After two and four days of CST, KEGG pathway enrichment analysis was performed to annotate DEGs in LJ11 and LJ25, with a q-value < 0.001. LJ11 had substantially more DEGs than LJ25, so there were more significantly enriched pathways in LJ11 than in LJ25. In total, 125 KEGG pathways were enriched in response to CS in the two rice varieties, while the Carbon metabolism pathway contained most DEGs (159) (Figure S2). Further analysis showed that thirty-three pathways were significantly enriched from the 12 parts of the Venn diagram, while no pathways were significantly enriched in the four parts within the threshold, which were 25 specific-4D, 11 specific-4U, common-2U, and common-4U (Fig. 4). The carbon metabolism pathway contained the most DEGs (77) that were significantly enriched in 11 specific-4D, followed by biosynthesis of amino acids (66 DEGs) in 11 specific-4D, plant hormone signal transduction (63 DEGs), phenylpropanoid biosynthesis (59 DEGs), and starch and sucrose metabolism (57 DEGs) in 11 specific-4U. The top three pathways with the fewest DEGs contained two, three, and four DEGs were monoterpenoid biosynthesis in common-2D, cutin, suberin, and wax biosynthesis in 25 vs. 11-4D, and ribosome biogenesis in eukaryotes in 25 specific-4U. Among these pathways, the cutin, suberin, and wax biosynthesis, photosynthesis-antenna proteins, and starch and sucrose metabolism were significantly enriched in three parts of the Venn diagram (Fig. 4 and Table S10). However, the plant hormone signal transduction pathway was considerably enriched in four Venn diagram sections. Thus we concentrated on this cascade.

### Potential functions of plant hormone signal transduction under CST

**Fig. 5:**
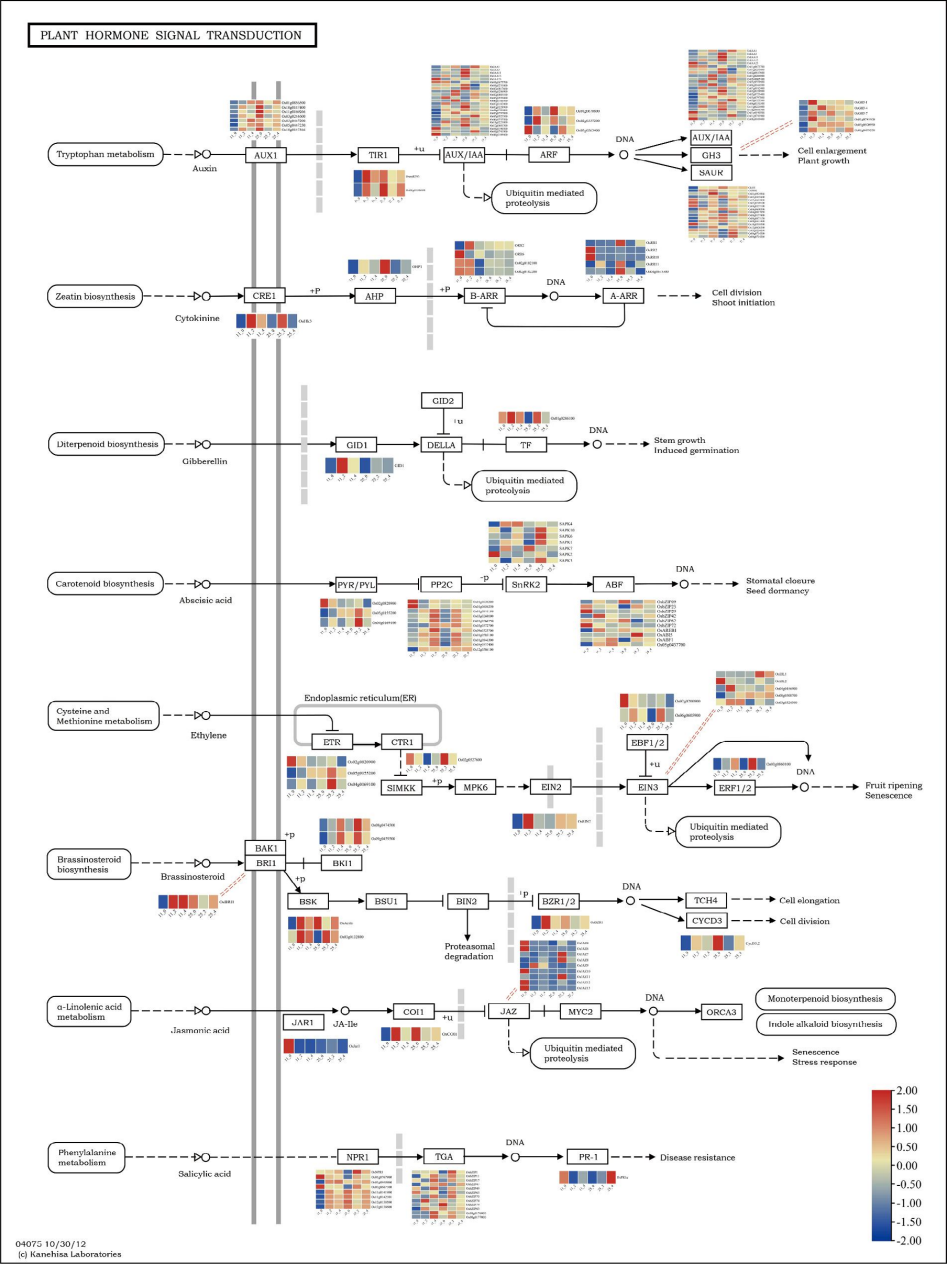
DEGs are involved in the plant hormone signal transduction pathway. The original figure of plant hormone signal transduction pathway was cited from the Kanehisa laboratories [97]. The heatmaps of DEGs represent the genes involved in the specific categories of the pathway. Gene IDs or gene symbols of the DEGs are listed on the heatmaps. The 11_0, 11_2, and 11_4 represent FPKM values in LJ11 under 0, 2, and 4 days of CST, respectively. The 25_0, 25_2, and 25_4 represent the FPKM values in LJ25 under 0, 2, and 4 days of CST, respectively

The Biocloud platform (http://www.biocloud.net/) identified 153 DEGs in the plant hormone signal transduction pathway from all comparing groups, involving eight plant hormones (Fig. 5 and Table S11). While, in the signal transduction of auxin, cytokinin (CK), gibberellin (GA), ABA, ETH, brassinosteroid (BR), JA, and SA, 58, 11, two, 31, 13, seven, 11, and 21 DEGs were identified, respectively. This indicated that auxin, ABA, SA, and ETH signaling pathways comprise the maximum number of DEGs, showing their importance in the rice reproductive response to CS. Similarly, according to the above mentioned order, the four plant hormone transduction pathways contained 52, 22, 15, and 6 DEGs in LJ11 and 22, 17, 14, and 5 DEGs in LJ25. The pathway’s largest segment, accounting for 37.9% of all DEGs, was involved in auxin signal transduction. Furthermore, 7, 2, 23, 3, 6, and 17 DEGs related to AUX1 (auxin transporter-like protein), TIR1 (leucine-rich repeats), AUX/IAA (AUX/IAA family), ARF (auxin response factor), GH3 (GH3 auxin-responsive promoter), and SAUR (auxin-responsive protein) were identified, respectively. As previously identified, genes, *OsIAA1*, *OsGH3-1*, *GLO3, ORR1, SAPK4*, and others, were all differentially expressed in LJ11 and LJ25.

In ABA transduction, 3, 11, 7, and 10 DEGs related to PYR/PYL (ABA receptor), PP2C (protein phosphatase 2 C), SnRK2 (Serine/threonine-protein kinase), and ABF (bZIP transcription factor) were detected, respectively. In LJ11 and LJ25, *SAPK4*, *SAPK6*, *OsbZIP09*, *OsbZIP29*, *OsABI5*, *OsABF1, OsbZIP23*, and other previously reported genes were all differently expressed.

In LJ11 and LJ25, the SA pathway was associated with eight NPR1 (regulatory protein NPR) related genes, twelve TGA (bZIP transcription factor) related genes, and one PR-1 (pathogenesis-related protein) linked gene. In the ETH transduction pathway, 3, 1, 1, 2, 5, and 1 DEGs, related to ETR (ETH receptor), CTR1 (serine/threonine-protein kinase), EIN2 (ETH-insensitive protein), EBF1/2 (EIN3-binding F-box protein 1), EIN3 (ETH-insensitive 3 protein), and ERF1/2 (ETH-responsive transcription factor) were identified, respectively.

### Functional network analysis of the DEGs in plant hormone signal transduction under CST

**Fig. 6:**
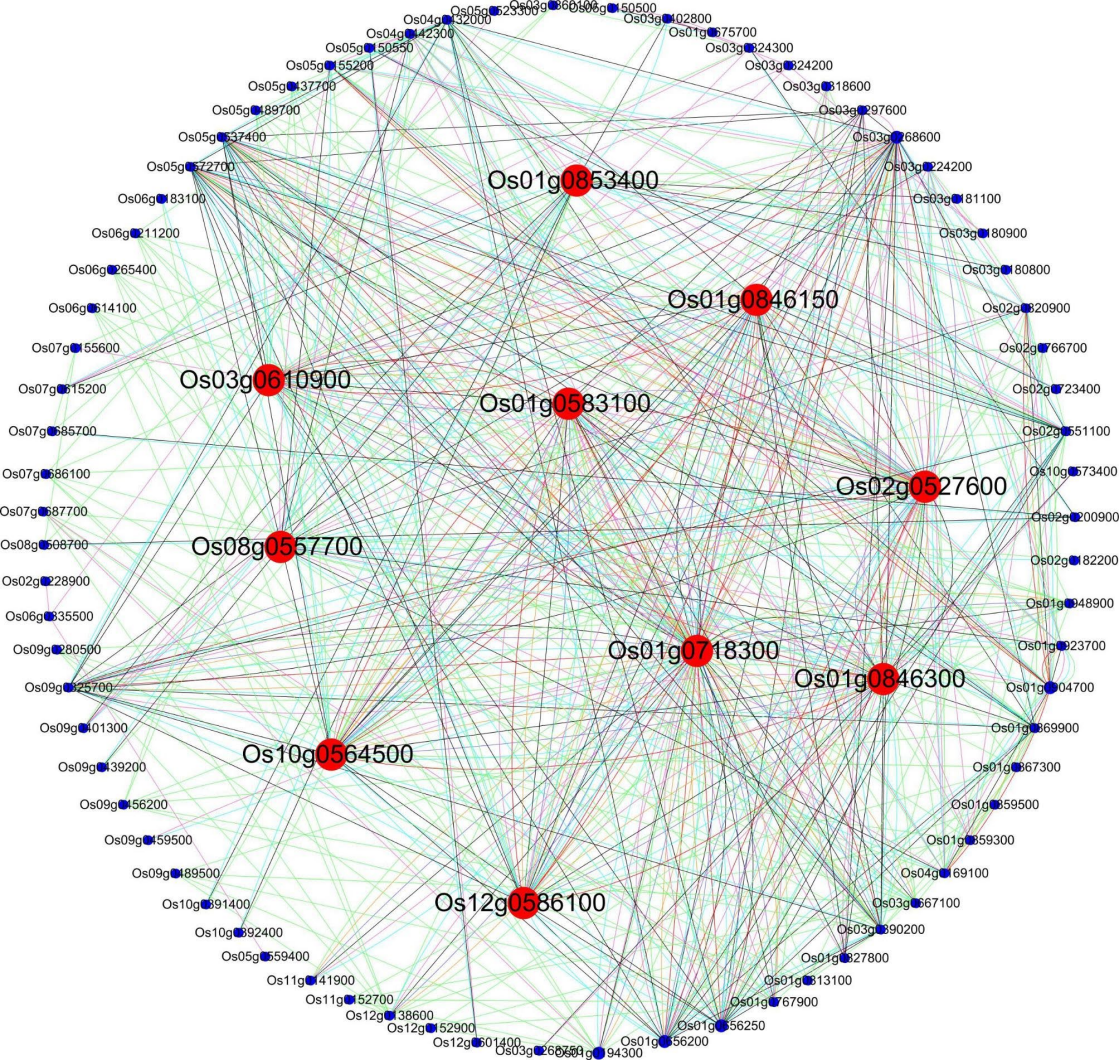
Interacting networks of DEGs in the KEGG pathway plant hormone signal transduction in LJ25 and LJ11 under different cold stress periods predicted by STRING and visualized in Cytoscape. The larger, red dots represent the hub genes. The small, blue dots represent other genes involved in interacting networks. Light green lines represent activation, orange lines represent expression, and pink lines represent binding. Purple lines represent catalysis, black lines represent inhibition, light blue lines represent post-translational modification (ptmod), and red lines represent reaction

A total of 153 DEGs implicated in the plant hormone signal transduction pathway were predicted using STRING (https://cn.string-db.org/), which was then visualized in Cytoscape. PPI analysis detected that 81 out of the 153 DEGs interacted with each other, and the number of co-expressed genes ranged from one to 76 (Fig. 6 and Table S12). The results showed that the ten genes related to protein phosphatase 2 C accounted for the highest proportion of the total number of PPI genes (12.35%), and most of those genes also had more co-expressed genes than other genes (ranging from two to 49). The DEGs in each of the nine categories co-expressed with other DEGs included nine primary region leucine zipper-related (ranging from two to nine), nine TIFY domain, eight transcription factor HBP-related (two to six), seven serine/threonine-protein kinase (one to three), seven AUX/IAA family-related (11 to 47), six response regulator receiver domain-related (one to seven), four ETH insensitive 3-related (two to 27), three abscisic acid receptor (three to five), and three NPR1/NIM1 like defense protein C-related, (two to six). Other categories with fewer DEGs were also detected (Table S12). Furthermore, with the threshold of co-expressed genes ≥ 30, ten DEGs were selected as hub genes, and *Os01g0718300* (*OsBRI1* and involved in the brassinosteroid transduction pathway) was the hub gene with the highest interaction degree interacting with 76 genes (Fig. 6; Table 4, S13).

**Table 4:** Annotation of the hub genes with co-expressed genes ≥ 30 identified in PPI analysis

#ID	plant hormone	category	gene symbol	hub gene number	annotation
Os01g0846150	abscisic acid	PP2C	NA	39	Protein phosphatase 2 C
Os01g0583100	abscisic acid	PP2C	NA	46	Protein phosphatase 2 C
Os01g0846300	abscisic acid	PP2C	NA	39	Protein phosphatase 2 C
Os12g0586100	abscisic acid	PP2C	SAPK9	47	Serine/threonine-protein kinase SAPK9
Os03g0610900	abscisic acid	SnRK	SAPK10	36	Serine/threonine-protein kinase SAPK10
Os10g0564500	abscisic acid	SnRK2	SAPK3	47	Serine/threonine-protein kinase SAPK3
Os01g0718300	brassinosteroid	BIR1	OsBRI1	76	Brassinosteroid LRR receptor kinase
Os08g0557700	cytokinine	AHP	OHP1	31	Histidine-containing phosphotransfer protein 1
Os02g0527600	ethylene	CTR1	NA	35	Serine/threonine-protein kinase CTR1
Os01g0853400	jasmonic acid	COI1	OsCOI1	30	Coronatine-insensitive protein 1

Note that ‘NA’ denotes a gene without a gene symbol

### qRT-PCR analysis for DEG validation

**Fig. 7:**
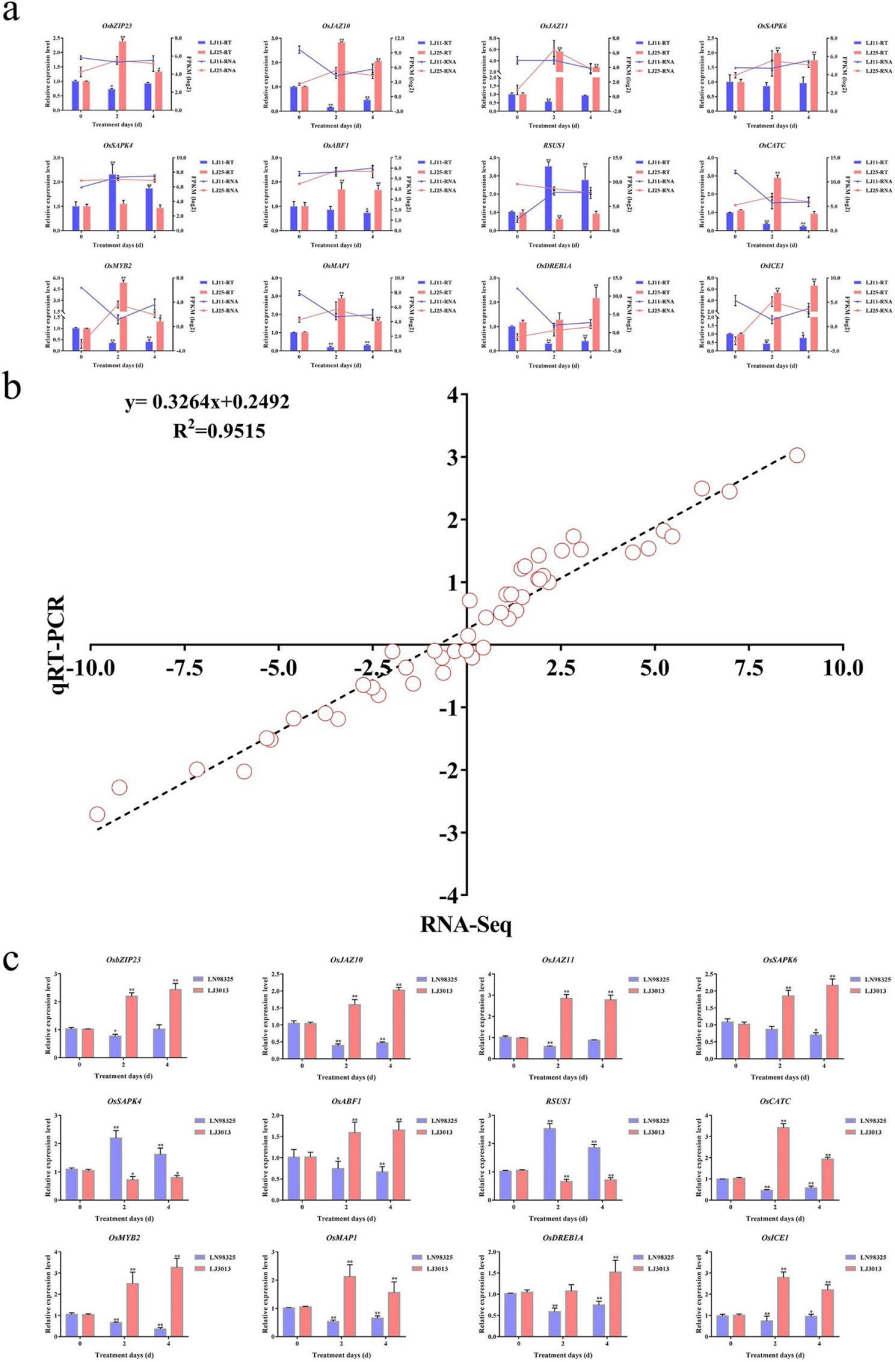
Quantitative real-time PCR validated the relative expression levels of DEGs related to 0, 2, or 4 days of CST at the transcriptional level in LJ11 and LJ25. (**a**) Expression levels of the twelve DEGs between LJ11 and LJ25. The line graphs indicate the log_2_FPKM of DEGs derived from RNA-Seq data, while the bar graphs represent the relative expression levels of DEGs determined from qRT-PCR validation after 0, 2, or 4 days of CST. (**b**) Results of correlation analysis between RNA-seq and qRT-PCR. The scatterplots are comprised of red circles that were formed from the log2 fold change of RNA-seq (y-axis) and the fold change of qRT-PCR (x-axis). The R^2^ (the correlation coefficient between qRT-PCR and RNA-seq) and the equation (the linear regression relationship) were presented. (**c**) The relative expression levels of DEGs in LN98325 and LJ3013 during the reproductive stage under 0, 2, or 4 days of CST. Values are presented as the means (± SD) of three biological replicates. * represent p < 0.05, ** represent p < 0.01. Error bars represent the SD

12 DEGs were selected for qRT-PCR based on the variations in expression patterns between LJ11 and LJ25 to validate the RNA-Seq results. Four of the six DEGs in the plant hormone signal transduction were engaged in ABA transduction (*OsABF1, OsSAPK4, OsSAPK6*, and *OsbZIP23*). The *OsJAZ10* and *OsJAZ11*, two DEGs, participated in JA. Six other DEGs were chosen as well, and these were *OsDREB1A*, *OsICE1*, *OsMYB2*, *OsMAP1*, *OsCATC*, and *RSUS1*. These DEGs had opposite patterns of expression. The results showed that most of the DEGs in the qRT-PCR analysis had similar expression patterns to the RNA-Seq results, albeit at different levels (Fig. 7a and b; Table S14). Furthermore, we confirmed the CS responsibility of these 12 DEGs through qRT-PCR analysis on another two rice varieties, which were LN98325 and LJ3013. LJ3013 is a CS resistant variety, and its EGRs was 40.88% after four days of CST, while, LN98325 is a CS susceptible one with EGRs of 64.90% (Figure S3, Table S15). Similarly, the findings of the qRT-PCR experiment on these two varieties revealed tendencies similar to those in LJ11 and LJ25 (Fig. 7c, Table S16). The qRT-PCR analysis generally confirmed the results of the RNA-Seq analysis, and the DEGs with potentially important effects in some molecular biological functions obtained from RNA-Seq could be further explored.

## Discussion

The CS has a wide range of complex impacts on rice growth and development as a significant environmental element of abiotic stress. The RNA-seq technique, based on microarray technologies, has been widely utilized to identify potential molecular genetics pathways globally under CS [46–48]. Several transcriptional studies on CST in rice have reported diverse results. This may be due to the differences in genotypes, duration of CST, or environmental conditions [33, 34, 49–52]. Moreover, most of the previous reports focused on *Indica* rice. In contrast, studies on the anthers in the *Japonica* rice in cold regions at the reproductive stage have rarely been reported [53].

### Relationship between the phenotypic characteristics and transcriptome results in the two varieties

The two *Japonica* rice varieties, LJ11 is highly susceptible to CS at the reproductive stage while LJ25 is the highly tolerant one [53]. This study confirmed that LJ25 has low EGRs and excellent CS resistance, while LJ11 has strong EGRs and weak CS resistance, consistent with earlier findings [53]. This was done by examining the EGRs of the two types. Additional pollen fertility research and another development observation further supported LJ25 and LJ11’s CS tolerance. In the current study, comparative transcriptome analysis was performed on the anthers of the two varieties in cold regions under CST. This study found 16,762 DEGs in total, which is similar to previous reports [54–56].

In addition, LJ11 had 11,956 and 11,275 DEGs after two and four days of CST, while LJ25 had 6,784 and 1,149 DEGs. There was no discernible difference in the amount of DEGs in LJ11 after two and four days of CST, indicating that LJ11 was more sensitive to CS and was continually severely impacted by it than that LJ25. The number of DEGs in LJ11 was much higher than that in LJ25. As a result, under CST, the phenotypic and transcriptome analyses revealed similar trends, indicating a strong and favorable connection between the two types of studies.

### Influences of CS on GO functional characterization between LJ25 and LJ11

The BP GO terms with KS < 0.001 in both LJ25 and LJ11 were identified and evaluated to clarify the changes in biological functions in plants under CS. Numerous up-regulated BP GO terms in LJ11 were associated with the floral organ development and morphogenesis, such as flower morphogenesis, microsporogenesis, mitotic nuclear division, nectary development, pollen development, male meiosis, and others. The findings imply that CS could boost LJ11’s growth process, which could be a stress response to CS while also reducing the negative impacts of CS. Plants can proliferate as a species by blossoming and generating progeny, even if they cannot survive as individuals in unfavorable environmental conditions [57]. Numerous studies have reported stress-induced flowering and provided much evidence [58, 59].

Additionally, plants exposed to CS risk significantly damaging their photosynthetic mechanism, impeding photosynthetic activity and depleting plant energy supplies [60]. In this study, the down-regulated GO terms in LJ11 primarily concentrated in the photosynthesis-related functional terms, such as photosynthesis, chloroplast organization, electron transport chain, and others, suggesting that these results were consistent with previous reports and LJ11 plants were severely affected by CS [53]. The substantially enriched up-and down-regulated GO terms in LJ25 were lower than in LJ11. Most of the terms were enriched after two days of CST and were related to the response to cold, response to heat, defense response to fungus, and others. This suggested that CS only affected LJ25 at the start of the CST and that it could gradually recover over the CST time until the end of processing. As a result, the CST had little effect on LJ25’s normal development and growth.

### Influences of CS on KEGG enrichment analysis between LJ25 and LJ11

Among the 33 significantly enriched KEGG pathways, 27 and 26 were annotated considerably after two and four days of CST, respectively. several down-regulated pathways related to photosynthesis were significantly enriched in LJ11, including photosynthesis, photosynthesis-antenna proteins, and carbon fixation in photosynthetic organisms, confirming that the photosynthesis in LJ11 was harmfully attacked by CS and again severely restricted energy supply for plant growth and development. Starch and sucrose metabolism was significantly enriched in three parts (25 specific-4U, 25 vs. 11-2U, and 25 vs. 11-4D), containing 140 DEGs. Starch is an indispensable energy supplier for pollen formation and pollen tube germination [61, 62]. Its accumulation could be disrupted by CS, resulting in a decrease in cell wall invertase activity and a reduction in sucrose concentration [11]. In this study, *OSINV4* was generally expressed in both LJ11 and LJ25 under CS. However, the *RSUS1*, *RSUS3*, *RSUS5*, and *RSUS6* (rice sucrose synthases encoding genes) [63] were almost up-regulated in LJ11 compared to LJ25, suggesting that some other regulators related to starch formation in pollen exist in rice in the cold regions. The *OsCIN1* and *GIF1*, two other genes that encode cell wall invertases, exhibit differential expression in grain filling and sucrose transport, respectively [64]. This shows that they may be major regulators of rice sucrose metabolism in cold regions under CS, which has not previously been documented.

### Influences of CS on the plant hormone signal transduction pathway

Plant hormones are pivotal regulators in plant growth and development [65]. The plant hormone signals are usually transmitted by linear signaling cascades of many signaling complexes [66]. Under CS, increased endogenous ABA of rice binds its PYP/PYL/RCAR receptor, suppressing PP2C activities and activating SnRK2, which could phosphorylate transcription factors and activate ABA-responsive genes, enhancing the CS-resistance [67]. *OsABF2* [68], a positive regulator of ABA signaling and drought tolerance of rice induced by many abiotic stresses, was up-regulated and normally expressed in LJ11 and LJ25, respectively, indicating it may be a positive regulator of CS of rice in the cold regions. *OsbZIP72* can bind ABRE to activate the expression of downstream reporter genes, whose overexpression could enhance the susceptibility to ABA and improve resistance to drought stress [69]. *OsABF1* (another ABF) has transcriptional activation activity, whose expression is induced by various abiotic stresses [44]. *OsbZIP72* was down-regulated in LJ11 and normally expressed in LJ25, while *OsABF1* was normally expressed in LJ11 and up-regulated in LJ25, suggesting they could be used for improving CS resistance. *OsSAPK6* and *OsSAPK1*, induced by osmotic stress [70], were both normally expressed in LJ11 and up-regulated in LJ25. The findings of this study indicate that these two genes may be activated by CS and play a significant role in the CS response. Additional studies are needed for further validation. Auxin is not only an essential hormone in plant growth and development but also participates in response to CS [71, 72]. In this study, most of the genes in the auxin signaling pathway (including *OsGH3.1* [73], *OsSAPK4* [74], and others) were up-regulated in LJ11 and customarily expressed in LJ25, suggesting that this pathway positively responds to CS in rice in the cold regions, similar to previous reports [75].

As the only gaseous hormone, ETH plays a versatile role in the response to CS [76, 77]. *EIN3* overexpression suppresses *CBFs* expression and leads to CS hypersensitivity in *Arabidopsis* [78]. In comparison, overexpression of ERF-related genes (*AtERF1* in *Arabidopsis* and *TaERF1* in wheat) can significantly enhance cold and drought resistance [79–81]. A total of 13 ETH-related genes, including *OsEIN2*, *OsEIL2*, and *OsEIL1*, were identified in this study, suggesting that the ETH signaling pathway is vital to the CS-response of rice in the cold regions.

The external application of CK enhances the CS resistance of *Arabidopsis* [82], while endogenous CK decreases under CS in rice and wheat [83, 84]. The A ARRs type genes play either positive or negative roles in CS-response in the CK signaling pathway [82]. Here, five of the 11 identified genes related to CKs were of type A ARR, including *OsRR2*, *OsRR11*, *OsRR1*, and *OsRR10.* Most of these genes were down-regulated in LJ25 compared to LJ11, suggesting that they play regulating roles in CS-response in the cold regions. Further research, however, is needed to confirm their positive or negative roles.

JA is involved in abiotic stress responses and inhibits plant growth under CS [85]. Similarly, external application of MeJA enhances CS and significantly up-regulates the expression of the *CBF* genes and the downstream *COR* gene [86]. The overexpression of *JAZ1* and *JAZ4* represses the expression of *CBFs* and the downstream genes, reducing the CS resistance [86]. Most of the *JAZ*-related genes were down-regulated in LJ11 and up-regulated in LJ25, implying that the JA pathway may play a positive role in CS-response in LJ11 compared to LJ25. However, the responses of SA and BR hormonal pathways to CS are primarily reported in *Arabidopsis* [87] and other crops, including cucumber [88], while the indicated study in rice is not well studied. In this study, 21 SA-related and seven BR-related genes were identified. Some genes were homologous to those in *Arabidopsis* involved in CS response, indicating that the genes in rice in the cold regions may play regulating roles in CS response but require further validation.

### The effect of hub genes involved in the PPI network in the CS response

Herein, ten hub genes were identified with the threshold of co-expressed genes ≥ 30. According to the findings, the ABA signaling system comprises six different genes, providing evidence that the stress hormone ABA plays a critical role in various harsh environmental conditions. Among the six ABA pathway-related genes, three were related to PP2C and three to SnRK (*SAPK9*, *SAPK10*, *SAPK3*) (Table S12). The SnRKs were positively regulated by the ABA signaling pathway, while PP2C is a negative regulator that inhibits SnRK2 kinase activity and represses ABA signal transduction [89]. In this study, PP2C related genes (*Os01g0846150*, *Os01g0583100*, and *Os01g0846300)* were co-expressed with 39, 46, and 39 genes, respectively. Most of which were up-regulated in LJ11 and generally expressed in LJ25. These results suggest that CS could enhance the expression of PP2C but does not significantly affect CS in LJ25. However, *SAPK9* [90], *SAPK10* [69, 70, 91], and SAPK3 [70, 92] co-expressed with 47, 36, and 47 genes, respectively, were negatively expressed in LJ11 under CS. *SAPK9* and *SAPK3* were positively expressed in LJ25 under CS, except for the normal expression of *SAPK10*, indicating that CS may partially enhance the expression of SnRKs in LJ25 compared to LJ11. Therefore, we speculate that the ABA signaling pathway was suppressed under LJ11 but activated in LJ25, inducing the CS response of the downstream genes. Moreover, these genes have not been previously reported to function in CS response and require further verification. The *OsCOI1* gene that encodes coronatine insensitive 1 was the only one involved in the JA signaling pathway and was co-expressed with 30 genes. As a component of JA signaling, *OsCOI1* can alter the protease inhibitor (TrypPI) content and the activity of peroxidase (POD) and polyphenol oxidase (PPO) [93]. In the current study, *OsCOI1* was identified to be up-regulated in LJ25 and generally expressed in LJ11, as not previously reported, indicating that *OsCOI1* was a positive regulator in response to CS in the cold regions. *OsBRI1* (*Os01g0718300*) is a BR receptor kinase encoding gene co-expressed with most genes (76 genes) in this study. These results suggest that it plays a pivotal role in response to CS. As in the BR signaling pathway, the membrane-localized receptor BRI1 and co-receptor BAK1 combine with BRs and then initiate the signaling network involving the expression of thousands of genes which ensure the growth regulation and stress response with BRs. The *OsBRI1* expression was induced in LJ25 but suppressed in LJ11. As a result, we hypothesize that the BRs signaling pathway may be activated by CS in cold regions and play a favorable function in response to CS. Therefore, more research is required to explore the underlying mechanism.

The expression patterns or levels of some DEGs were verified by qRT-PCR analysis, which preliminarily confirmed that these genes played positive roles in the response to CS in rice and could be treated as candidate genes for regulating CS resistance. However, to understand how these genes contribute to the CS response and to identify the real CS response genes, further verification and functional analysis of these genes need to be carried out. Therefore, in future studies, the function of these genes in response to CS in rice will be explored by generating transgenic plants with overexpressing and CRISPR-Cas9 genome editing of them.

## Conclusion

A comprehensive analysis of anther gene expression using parallel RNA-seq revealed significant differences in genetic adaptation to CS and globally varied transcription reprogramming between LJ11 and LJ25 during the reproductive stage. Firstly, LJ11 demonstrated increased susceptibility to CS with greater DEGs than LJ25. Secondly, GO enrichment analysis showed that most photosynthesis-related GO terms were down-regulated in LJ11 compared with LJ25. The GO terms ‘response to cold’ and ‘response to heat’ were the most enriched. Thirdly, KEGG enrichment analysis showed critical metabolic and regulatory related pathways, particularly the KEGG pathway ‘plant hormone signal transduction pathway’. Finally, we identified numerous hub genes in response to CS using the PPI network on the DEGs in this pathway. Several DEGs, including *OsDREB1A*, *OsICE1*, *OsMYB2*, *OsABF1*, *OsCATC*, and others, showed distinct expression patterns among the rice varieties (Table 5), indicating that they are primarily accountable for CS resistance and require further validation(such as transgenic and gene editing methods)for possible future use in CS-resistant molecular breeding of rice in cold regions.

**Table 5:** List of candidate genes in response to CS at the reproductive stage of rice

Gene symbol	11-0-2_FDR	11-0-2_log2FC	11-0-4_FDR	11-0-4_log2FC	25-0-2_FDR	25-0-2_log2FC	25-0-4_FDR	25-0-4_log2FC	Annotation
OsJAZ11	6.36E-05	-2.35	7.39E-02	-0.86	3.29E-07	6.25	6.70E-04	3.03	tify domain
OsJAZ10	1.27E-04	-5.22	2.44E-03	-3.76	6.11E-08	2.53	1.52E-03	1.94	tify domain
OsbZIP23	1.29E-02	-0.63	2.20E-01	-0.32	2.51E-07	1.54	3.46E-01	1.11	bZIP transcription factor
OsDREB1A	6.85E-105	-9.82	1.08E-85	-9.23	1.36E-02	1.91	1.00E-03	2.84	AP2 domain
OsICE1	8.42E-04	-4.60	1.35E-01	-1.62	4.36E-103	8.78	2.76E-10	5.46	Transcription factor ICE1
OsMYB2	6.03E-61	-5.32	2.29E-21	-2.51	7.03E-27	6.99	7.97E-07	4.83	Myb-like DNA-binding domain
OsMAP1	1.37E-12	-3.42	1.34E-09	-2.75	5.18E-10	1.90	9.27E-01	0.08	Protein tyrosine kinase
OsCATC	1.26E-40	-7.17	4.82E-31	-5.91	2.45E-12	2.03	3.34E-01	0.91	Catalase
RSUS1	4.47E-91	5.23	8.36E-16	4.41	1.75E-03	-1.42	9.93E-04	-1.98	Sucrose synthase

## Methods

### Plant materials: growth conditions and CS treatment

LJ11 and LJ25 were provided by the Rice Research Institute of Heilongjiang Academy of Agricultural Sciences (Jiamusi, China). After surface-sterilization and incubation at 32 °C for two days, the germinated seeds were sown in pots. Twenty seeds were sown in each pot in a ring. Three pots each represented biological replicates for both the CS treatment (CST) and control groups were grown in a greenhouse at 28/22°C (day/night) with 80% relative humidity (RH) and a 14-hour photoperiod. Following Satake and Hayase’s [94] methodology, the CST was performed when the auricles of the flag leaf were around − 5 − 0 cm below the penultimate leaf, with the pollen thought to be through meiosis, the most susceptible stage to CS [94, 95]. The CST groups in this stage were moved into a growth chamber (Beijing ZNYT, China) at 12 °C (± 0.5 °C) with a 12-h light/dark photoperiod and 75–85% RH for four days and then returned to the original chamber until maturity. Cold resistance was evaluated based on the empty grains of the spikelet per plant. SigmaPlot software version 12.5 (Systat Software Inc., San Jose, CA, USA) was used for variance analysis. The mean differences (p < 0.05) were compared with Duncan’s multiple range test.

### Pollen fertility examination

After zero, two, and four days of CST, the anthers of LJ11 and LJ25 were collected from spikelets before flowering for pollen fertility examination. After crushing and removing the anther wall on a glass slide, the pollen grains were stained with 1% potassium iodide solution (I_2_-KI) and photographed with a Leica DFC300 microscope. The pollen morphology and degree of staining were used to determine the maturity and fertility of the pollen grains. The fertile pollen grains are round and dark brown, while sterile pollen grains are unstained, yellow, and irregularly shaped.

### Cytological observation

The anthers of LJ11 and LJ25 at different CST (zero, two, and four days) were collected and fixed in FAA solution for 48 h at room temperature. The tissues were then dehydrated with a graded alcohol series (75, 85, 90, 95, and 100%). The implanted tissues were sectioned at 4 to 6 μm thickness using a microtome (Leica RM2235) after xylene washing and paraffin infiltration and after being dried at 60 °C to eliminate water and the wax was melted. The sections were stored at room temperature. After staining with 0.5% toluidine blue (m/v), the anther sections were observed and captured using a microscope (Nikon Eclipse 80i).

### RNA isolation, sequencing, and library preparation

In the CST groups, anther samples of LJ11 and LJ25 were collected from florets (3–5 mm length) in the upper third of each panicle in the same position and incubated at 12 °C for zero day, two days, or four days. The anthers collected at 0 days were treated as control. Each sample was collected in triplicate, flash-frozen in liquid nitrogen, and stored at -80 °C until future use. total mRNA was extracted with XcelGen total RNA isolation kit (Xcelris Genomics, India) according to the manufacturer’s instructions. The RNA quantity and quality were assessed using a Nanodrop 8000 Spectrophotometer (Thermo Scientific, USA) based on RNA absorbance at 260 and 280 nm.

Furthermore, RNA 6000 Nano LabChip (Agilent Technologies, USA) was used to examine the RNA integrity using the Agilent Bioanalyzer 2100 (Agilent Technologies, USA). The sequencing library of each sample was constructed according to the NEBNext Ultra Directional RNA library prep kit protocols for Illumina (NEB, USA). After purification, the NEBNext First-Strand Synthesis Reaction Buffer was used to fragment mRNA at a higher temperature. Based on random hexamer primer and M-MuLV reverse transcriptase, the first-strand cDNA was first produced. Then, using DNA polymerase I and RNase H, the second strand of the cDNA was synthesized, and finally, it was adaptor-ligated. After gel electrophoresis and PCR amplification, the cDNA fragments (200–250 bp) were selected for RNA-seq on an Illumina Hiseq™2500 platform. The paired-end (2 × 150 bp) reads were generated following the manufacturer’s protocols (Beijing Biomarker Biotechnology Co., Beijing, China).

### RNA-seq data analysis

Raw reads were first generated in fastq format after sequencing. After filtering adaptors, low-quality reads (more than 50% bases with Phred quality score ≤ 5) and poly-N (≥ 1%), Q20, Q30, GC content, and sequence duplication level of the clean data were calculated to ensure the downstream analyses performed on high-quality data. Using HISAT2 (v2.1.0) with the default settings, clean reads were successfully mapped to the reference genome of *Oryza sativa* L. ssp. *Japonica* (Oryza sativa IRGSP-1.0). HTSwq package (v0.6.0) was applied to count the number of reads mapped to genes. FPKM (Fragments Per Kilobase of transcript per Million mapped reads) was used to estimate the gene expression levels. Genes with read counts less than ten were identified as having low expression and excluded from differential gene expression analysis. Using the DESeq2 (v1.6.3), differential expression analysis of two groups was carried out [96]. Fold change (FC) ≥ 2 and P-value ≤ 0.05 were chosen as the identification thresholds for DEGs.

### Functional annotation of the DEGs

Blast2GO (v2.5) was used for Gene Ontology (GO) annotation based on the non-redundant (Nr) nucleotide and protein databases in the National Center for Biotechnology Information (NCBI, https://www.ncbi.nlm.nih.gov/). The GO terms were significantly enriched with the corrected p-value (Kolmogorov–Smirnov; KS) ≤ 0.01. The DEGs were mapped to the Kyoto Encyclopedia of Genes and Genomes database (KEGG; http://www.genome.jp/kegg) for pathway enrichment analysis using the clusterProfiler package (http://bioconductor.org/packages/release/bioc/html/clusterProfiler.html) [97]. CummeRbund (an R package) was used to construct the heat maps that illustrate the DEGs [76]. The PPI network analysis of the DEGs was performed with RiceNet (v2.0) (https://www.inetbio.org/ricenet/) [98], followed by visualized through Cytoscape (http://www.cytoscape.org). Genes and their interactions were represented by nodes and edges (links) [99].

### Validation by quantitative real-time PCR

A total of 12 DEGs from the plant signaling pathway were selected to verify the RNA-seq results’ accuracy through qRT-PCR (quantitative reverse-transcription polymerase chain reaction) (Table S1). The specific primers to the target genes were designed with Primer3 (https://primer3.ut.ee/) and Primer-BLAST on the NCBI (Table S2). The cDNA synthesis, RT-PCR, and data analysis were performed as previously reported [16].

## Electronic supplementary material

Below is the link to the electronic supplementary material.

Supplementary Material 1

Supplementary Material 2

Supplementary Material 3

Supplementary Material 4

Supplementary Material 5

Supplementary Material 6

Supplementary Material 7

Supplementary Material 8

Supplementary Material 9

Supplementary Material 10

Supplementary Material 11

Supplementary Material 12

Supplementary Material 13

Supplementary Material 14

Supplementary Material 15

Supplementary Material 16

Supplementary Material 17

Supplementary Material 18

Supplementary Material 19

## Data Availability

All data generated or analyzed during this study are included in this published article. Raw sequence data were deposited in the Sequence Read Archive (SRA) database (www.ncbi.nlm.nih.gov/sra), Bio BioProject Accession: PRJNA495106 (www.ncbi.nlm.nih.gov/bioproject).

## Abbreviations

**CS**: Cold stress
**LJ11**: Longjing11
**LJ25**: Longjing25
**DEGs**: Differentially expressed genes
**GO**: Gene ontology
**KEGG**: Kyoto Encyclopedia of Genes and Genomes
**ABA**: Abscisic acid
**SA**: Salicylic acid
**ETH**: Ethylene
**PPI**: Protein-protein interaction
**ROS**: Reactive oxygen species
**IAA**: Indoleacetic acid
**JA**: Jasmonic acid
**CST**: CS treatment
**RH**: Relative humidity
**CK**: Cytokinin
**GA**: Gibberellin
**BR**: Brassinosteroid
