# Supplementary figures and images for: Comparison of anther transcriptomes in response to cold stress at the reproductive stage between susceptible and resistant Japonica rice varieties
Source: BMC Plant Biol. 2022 Oct 26;22:500. doi: 10.1186/s12870-022-03873-6 (PMC9597962; doi:10.1186/s12870-022-03873-6)

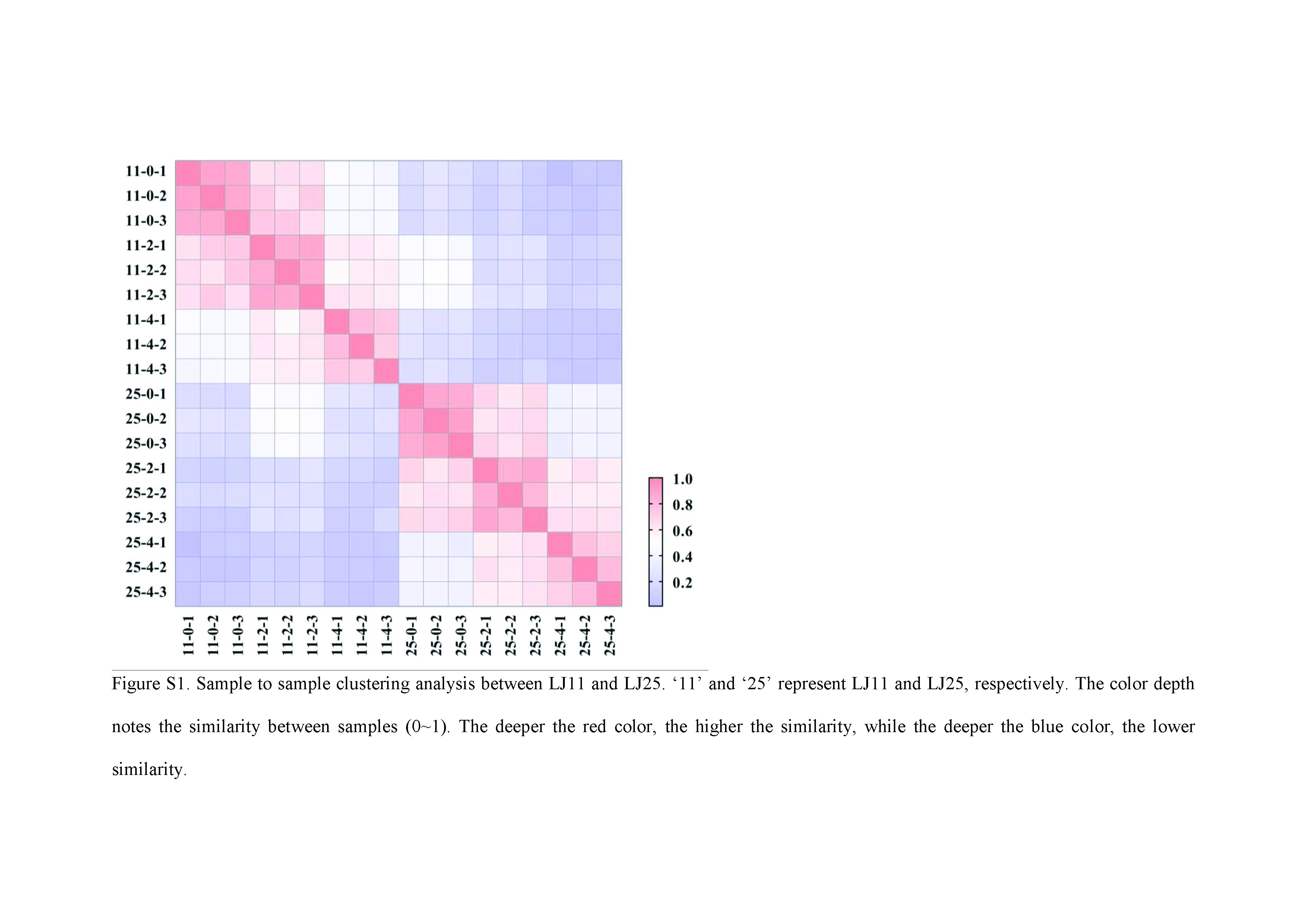

Supplement: Supplementary file 17 — Supplementary Material 17 [file 12870_2022_3873_MOESM17_ESM.jpg]

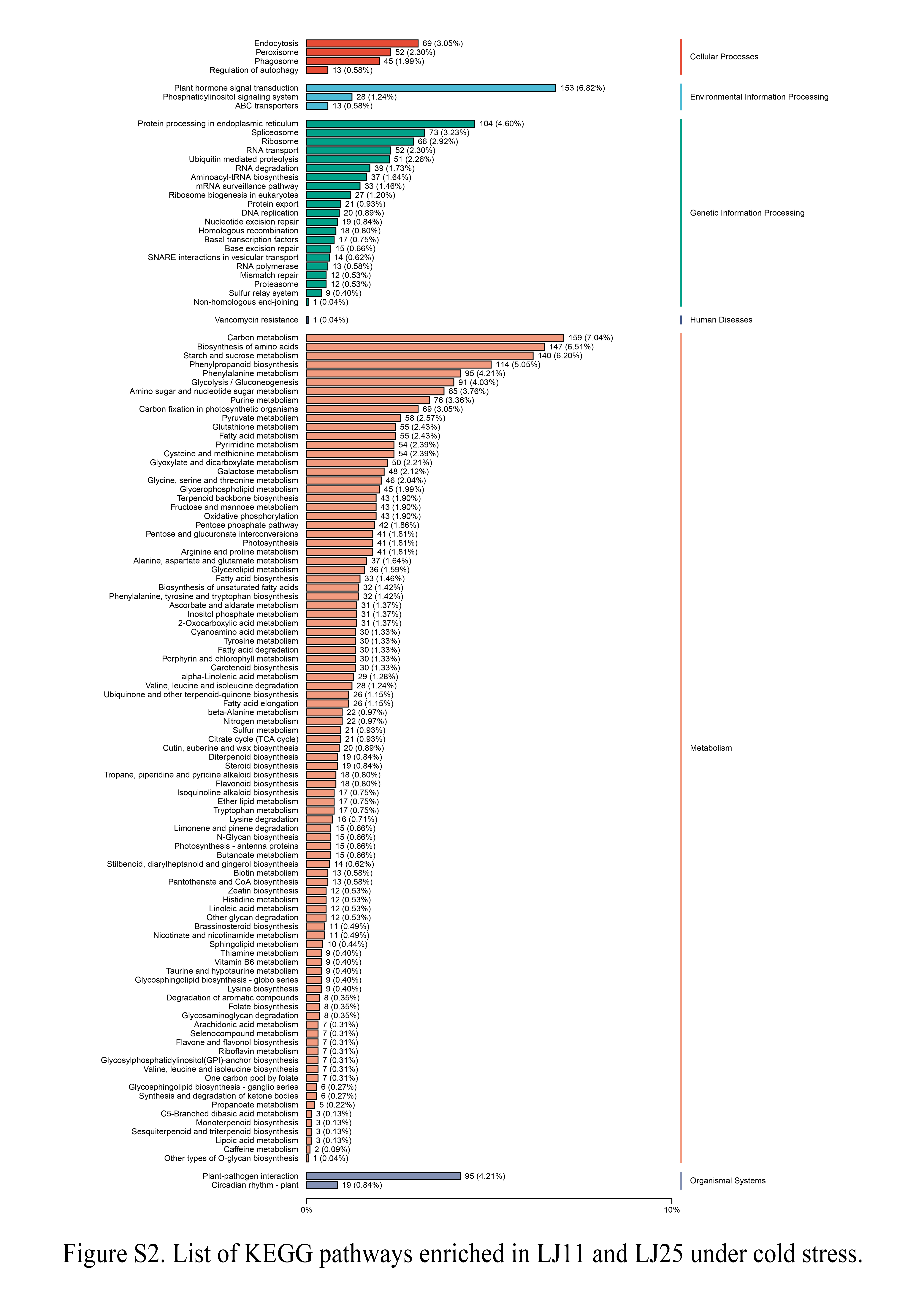

Supplement: Supplementary file 18 — Supplementary Material 18 [file 12870_2022_3873_MOESM18_ESM.jpg]

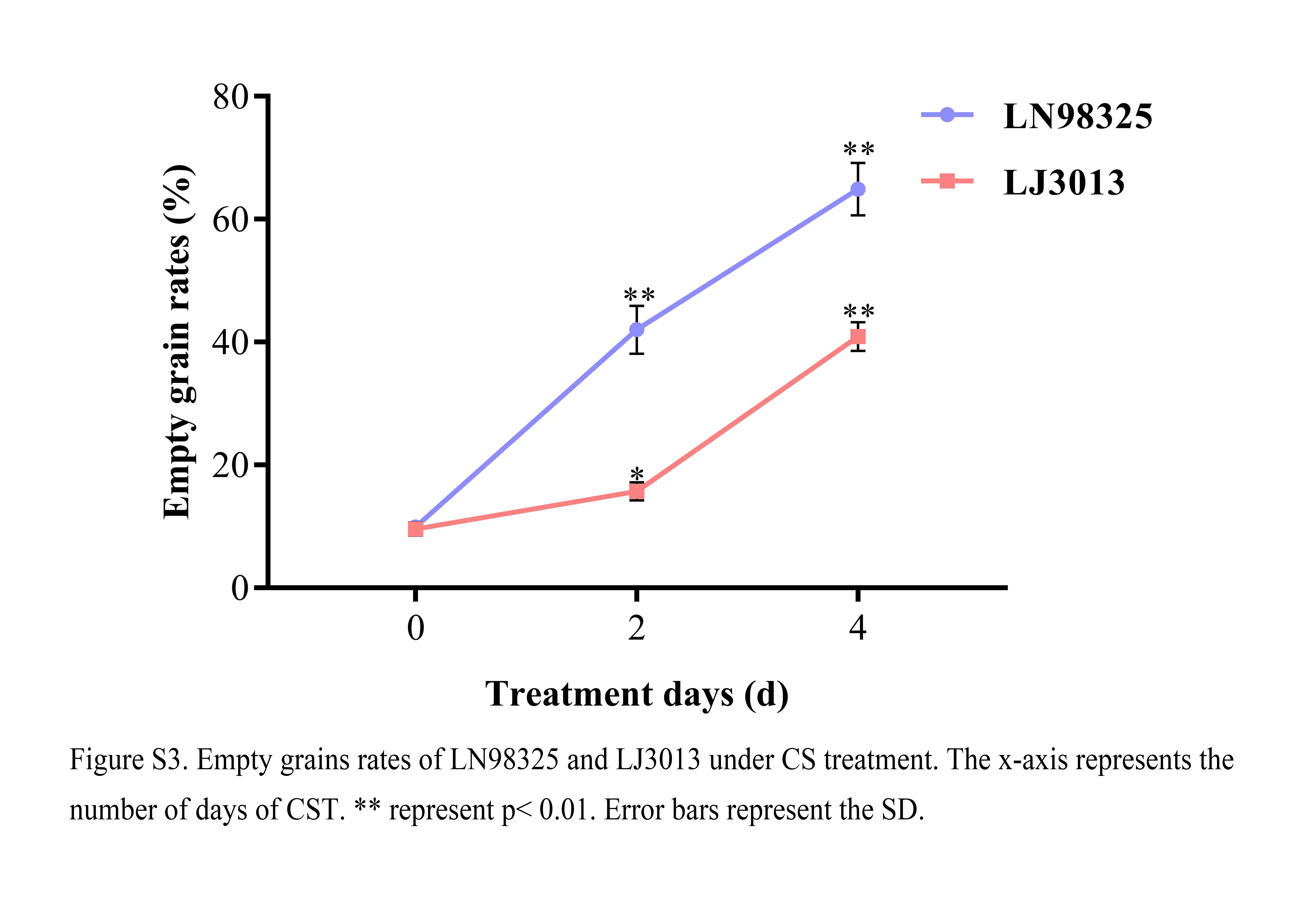

Supplement: Supplementary file 19 — Supplementary Material 19 [file 12870_2022_3873_MOESM19_ESM.jpg]
